# Evaluation of ^89^Zr-Labeled Human Anti-CD147 Monoclonal Antibody as a Positron Emission Tomography Probe in a Mouse Model of Pancreatic Cancer

**DOI:** 10.1371/journal.pone.0061230

**Published:** 2013-04-05

**Authors:** Aya Sugyo, Atsushi B. Tsuji, Hitomi Sudo, Kotaro Nagatsu, Mitsuru Koizumi, Yoshinori Ukai, Gene Kurosawa, Ming-Rong Zhang, Yoshikazu Kurosawa, Tsuneo Saga

**Affiliations:** 1 Diagnostic Imaging Group, Molecular Imaging Center, National Institute of Radiological Sciences, Chiba, Japan; 2 Molecular Probe Program, Molecular Imaging Center, National Institute of Radiological Sciences, Chiba, Japan; 3 Division of Antibody Project, School of Medicine, Fujita Health University, Toyoake, Japan; Genentech, United States of America

## Abstract

**Introduction:**

Pancreatic cancer is an aggressive cancer and its prognosis remains poor. Therefore, additional effective therapy is required to augment and/or complement current therapy. CD147, high expression in pancreatic cancer, is involved in the metastatic process and is considered a good candidate for targeted therapy. CD147-specfic imaging could be useful for selection of appropriate patients. Therefore, we evaluated the potential of a fully human anti-CD147 monoclonal antibody 059-053 as a new positron emission tomography (PET) probe for pancreatic cancer.

**Methods:**

CD147 expression was evaluated in four pancreatic cancer cell lines (MIA Paca-2, PANC-1, BxPC-3, and AsPC-1) and a mouse cell line A4 as a negative control. Cell binding, competitive inhibition and internalization assays were conducted with ^125^I-, ^67^Ga-, or ^89^Zr-labeled 059-053. *In vivo* biodistribution of ^125^I- or ^89^Zr-labeled 059-053 was conducted in mice bearing MIA Paca-2 and A4 tumors. PET imaging with [^89^Zr]059-053 was conducted in subcutaneous and orthotopic tumor mouse models.

**Results:**

Among four pancreatic cancer cell lines, MIA Paca-2 cells showed the highest expression of CD147, while A4 cells had no expression. Immunohistochemical staining showed that MIA Paca-2 xenografts also highly expressed CD147 *in vivo*. Radiolabeled 059-053 specifically bound to MIA Paca-2 cells with high affinity, but not to A4. [^89^Zr]059-053 uptake in MIA Paca-2 tumors increased with time from 11.0±1.3% injected dose per gram (ID/g) at day 1 to 16.9±3.2% ID/g at day 6, while [^125^I]059-053 uptake was relatively low and decreased with time, suggesting that 059-053 was internalized into tumor cells *in vivo* and ^125^I was released from the cells. PET with [^89^Zr]059-053 clearly visualized subcutaneous and orthotopic tumors.

**Conclusion:**

[^89^Zr]059-053 is a promising PET probe for imaging CD147 expression in pancreatic cancer and has the potential to select appropriate patients with CD147-expressing tumors who could gain benefit from anti-CD147 therapy.

## Introduction

Pancreatic cancer is a commonly diagnosed cancer and the eighth leading cause of cancer death worldwide, accounting for 278,684 of estimated new cancer cases and 266,669 of estimated cancer deaths [1] (GLOBOCAN 2008, http://globocan.iarc.fr/). Pancreatic cancer patients present minor symptoms at medical evaluation, and the silent nature of this disease that is not apparent until late in the disease process contributes to a very poor prognosis. Only 7% of patients present with localized, potentially curable tumors at diagnosis and approximately 50% of pancreatic cancer patients are diagnosed at advanced stages of disease [2]. The overall 5-year survival rate among patients with pancreatic cancer is 6% in the United States [2]. Therefore, additional effective anticancer therapy is required to augment and/or complement the present treatment strategies such as surgery and chemo/radiotherapy, especially for patients with metastatic cancer.

CD147 (so-called EMMPRIN) is a 55-kDa transmembrane protein of the immunoglobulin superfamily and is involved in many physiological functions, such as spermatogenesis, embryo implantation, lymphocyte activation, neural network formation at early stages and induction of monocarboxylate transporters [3]. CD147 expresses in many types of tumors including pancreatic cancer [4]. CD147 induces expression of matrix metalloproteinases (MMPs), such as MMP-1, MMP-2, MMP-9, MT1-MMP, and vascular endothelial growth factor. Overexpression of CD147 in breast cancer cells by expression vector transfection resulted in increased tumor growth and metastasis [5]. These findings suggest that CD147 is involved in invasion, metastasis, angiogenesis and tumor proliferation, and therefore is a good candidate for targeted cancer therapy. Depletion of CD147 by RNA interference or specific antibody reduced the proliferation, invasion, metastasis of tumors and blood vessel formation, and therefore clinical trials of CD147-targeted therapy have been conducted [3], [6], [7]. Although the incidence of CD147 expression is high (87%) in pancreatic cancer [4], some tumors do not express CD147, and thus are not suitable candidates for CD147-targeted therapy. It is therefore important to use a noninvasive imaging method to evaluate the CD147 status in an individual tumor at the time of treatment planning to select appropriate patients for CD147-targeted therapy.

Recently, we isolated a novel fully human monoclonal IgG_1_ antibody designated as 059-053 against CD147 from a large-scale human antibody library constructed using a phage-display system that incorporated a highly efficient screening method termed isolation of antigen–antibody complexes through organic solvent, with living pancreatic cancer cells [8]. This antibody induces antibody-dependent cell-mediated cytotoxicity (ADCC) and inhibits cell proliferation of pancreatic cancer cells [8], [9]. In the present study, we radiolabeled 059-053, and evaluated the *in vitro* and *in vivo* properties as a new positron emission tomography (PET) probe for imaging CD147-expressing tumors in a pancreatic cancer model.

## Materials and Methods

### Cells

Human pancreatic cancer cell lines (MIA Paca-2, PANC-1, BxPC-3, and AsPC-1) were obtained from American Type Culture Collection (Manassas, VA, USA). A4 cells, established from mouse 3T3 cells transfected with a human HER2-expression vector [10], were used as a negative control. The cells were maintained in RPMI 1640 medium (Sigma, St. Louis, MO, USA) supplemented with 5% fetal bovine serum (Sigma) in a humidified incubator maintained at 37°C with 5% CO_2_.

### Subcutaneous and orthotopic tumor mouse models

The animal experimental protocol was approved by the Animal Care and Use Committee of the National Institute of Radiological Sciences, and all animal experiments were conducted in accordance with the institutional guidelines regarding animal care and handling. BALB/c-nu/nu male mice (5 weeks old, CLEA Japan, Tokyo, Japan) were maintained under specific pathogen-free conditions. To induce a subcutaneous tumor model, mice were inoculated subcutaneously with MIA Paca-2 (4×10^6^) and A4 (1×10^6^) cells in the left and right thighs, respectively, under isoflurane anesthesia. Since A4 cells grow faster than MIA Paca-2 cells in mice, the inoculation day of each cell line was adjusted to ensure that tumor xenografts were of equal size at the time of experiment (approximately 30-day interval between the two inoculations). To induce an orthotopic tumor model, we surgically implanted a xenografted tumor in the pancreas as previously described [11]. Briefly, a subcutaneous tumor was resected aseptically, necrotic tissues were removed, and the remaining viable tumor tissues were minced into pieces of approximately 5 mm in diameter in PBS (Sigma). Recipient mice were anesthetized with isoflurane inhalation, the skin and abdominal wall were incised above the pancreas, the pancreas was carefully exposed, and a tumor piece was transplanted onto the tail of the pancreas using a 6-0 silk suture (Alfresa Pharma, Osaka, Japan). The pancreas was returned to the peritoneal cavity, and the skin and abdominal wall were closed with a 6-0 silk suture.

### CD147 protein expression analysis

Western blotting and immunofluorescence staining were conducted as previously described [12], [13]. Briefly, for western blotting, the cell lysate was resolved by sodium dodecyl sulfate polyacrylamide gel electrophoresis, transferred to a polyvinylidene fluoride (PVDF) membrane (Hybond-P, GE Healthcare, Little Chalfont, UK) and reacted with a rabbit anti-CD147 monoclonal antibody (EPR4053, Abcam, Cambridge, UK) for 1 h at room temperature. The primary antibody was reacted with a horseradish peroxidase-linked anti-rabbit antibody (GE Healthcare) and the membrane was visualized using an ECL Plus kit (GE Healthcare). After images were captured using a LAS-3000 imaging system (FujiFilm, Tokyo, Japan), antibodies on the PVDF membrane were removed with stripping buffer and stained the membrane with Coomassie Brilliant Blue (ATTO, Tokyo, Japan) as a loading control. The intensity of each band was quantified using ImageJ software (National Institute of Mental Health, Bethesda, MD, USA). For immunofluorescence staining, cells were grown on glass coverslips and fixed in cold methanol for 5 min. Nonspecific binding of the antibody was blocked by applying Block Ace reagent (Dainippon Pharmaceutical, Osaka, Japan) with 10% goat serum for 30 min. Cells were incubated with 059-053 as the primary antibody [8] overnight at 4°C. A secondary anti-human antibody conjugated with Cy3 (Jackson ImmunoResearch Laboratories, West Grove, PA, USA) was applied for 30 min at room temperature. Nuclei were stained with DAPI in mounting medium. The images were obtained with an exposure time of 500 ms for CD147 using a fluorescence microscope (Olympus, Tokyo, Japan). The intensity of CD147 staining in each cell line was quantified using ImageJ software. For immunohistochemical staining, mice bearing subcutaneous tumors (MIA Paca-2 and A4) were euthanized, and tumors were removed and quickly frozen in optimal-cutting-temperature compound (Sakura Finetek Japan, Tokyo, Japan). Dried sections (10 µm thick) were fixed with cold methanol and were immunostained with goat anti-CD147 antibody (AF972, R&D Systems, Minneapolis, MN, USA). Nonspecific binding was blocked by Block Ace regent with 10% goat serum. The specimens were incubated for 1 h at room temperature with the primary antibody. A peroxidase-conjugated secondary antibody (Simple Stain MAX-PO, Nichirei Biosciences, Tokyo, Japan) was applied for 30 min at room temperature and then visualized with a diaminobenzidine staining reagent (Simple Stain DAB solution, Nichirei Biosciences).

### Radiolabeling of antibody

For ^67^Ga and ^89^Zr labeling, the human anti-CD147 monoclonal antibody 059-053 (IgG_1_) [8] was conjugated with *p*-isothiocyanatobenzyl-desferrioxamine B (DF; Macrocyclics, Dallas, TX, USA) at DF to antibody molar ratio of 3∶1, as previously described [14]. The conjugation ratio of DF to antibody was estimated to be 1.0 to 1.3 from ^67^Ga-DF-conjugated antibody to ^67^Ga-DF ratio determined by size exclusion chromatography using a PD10 column (GE Healthcare) before purification. Non-conjugated chelate was removed using a Sephadex G-50 (GE Healthcare) spin column. For ^67^Ga labeling, the DF-conjugated antibody (45 µg in 10 µL PBS) was incubated with 600 kBq of ^67^Ga-chloride (70–80 MBq/mL, Nihon Medi-Physics, Tokyo, Japan) for 1 h at room temperature, and radiolabeled antibodies were purified on a Sephadex G-50 spin column. The radiochemical yield of ^67^Ga-labeled antibody was 45% to 63%, the radiochemical purity was greater than 96%, and the specific activity was 6 to 8 kBq/ µg determined by PD10 column chromatography. For ^89^Zr labeling, ^89^Zr was produced by a (p,n) reaction on ^89^Y (New Metals and Chemicals, Waltham Abbey, UK) in an alumina ceramic vessel (Kyocera, Kyoto, Japan) by vertical irradiation using the NIRS AVF-930 cyclotron and purified with a hydroxamate column by an automated recovery/purification apparatus as previously described [15]. The DF-conjugated antibody (100 µg in 20 µL PBS) was incubated with 2.8 to 5.2 MBq of ^89^Zr-oxalate (3.7–5.6 GBq/mL in 1 M oxalate, pH 7–8) for 1 h at room temperature, and radiolabeled antibodies were purified on a Sephadex G-50 spin column. The radiochemical yield of ^89^Zr-labeled antibody was 54% to 86%, the radiochemical purity was greater than 96%, and the specific activity was 16 to 43 kBq/ µg determined by thin-layer chromatography using 50 mM diethylenetriaminepentaacetic acid (Sigma, pH 7) as the mobile phase. ^125^I labeling was conducted with Na^125^I (PerkinElmer, Waltham, MA, USA) and chloramine-T (Wako Pure Chemical Industries, Osaka, Japan) as previously described [12], resulting in specific activities ranging from 119 to 669 kBq/ µg.

### *In vitro* assay

Cell binding, competitive inhibition, and internalization assays were conducted as previously described [16]. Briefly, in a cell binding assay, serially-diluted MIA Paca-2 or A4 cells in PBS with 1% BSA (Sigma) were incubated with the radiolabeled antibody on ice for 60 min. After washing, the radioactivity bound to the cells was measured. The immunoreactivity of radiolabeled antibodies was estimated according to the method of Lindmo *et al*. [17]. In a competitive inhibition assay, the radiolabeled antibody was incubated with MIA Paca-2 cells in the presence of varying concentrations of the unlabeled antibody on ice for 60 min. After washing, radioactivity bound to the cells was counted. The dissociation constant was estimated by applying data to a one-site competitive binding model using GraphPad Prism software (Graphpad Software, La Jolla, CA, USA). In an internalization assay, MIA Paca-2 cells were preincubated in culture medium with ^125^I- or ^67^Ga-labeled antibody on ice for 60 min. After washing, collected cells were further cultured at 37°C or on ice in fresh medium without radiolabeled antibodies. At various time points, the supernatant and the cells were separated by centrifugation. Trichloroacetic acid was added to the supernatant on ice and then separated by centrifugation to determine the non-protein-bound fraction (supernatant) and protein-bound fraction (pellet). The cells were washed with acidic buffer and then separated by centrifugation to determine both the membrane-bound fraction (supernatant) and internalized fraction (pellet). We conducted these *in vitro* assays in duplicate.

### Biodistribution

When subcutaneous tumors reached a diameter of approximately 10 mm, mice were intravenously injected with the mixture of ^89^Zr- and ^125^I-labeled antibody (37 kBq each). The total injected protein dose was adjusted to 20 µg per mouse by the addition of unlabeled antibody. At 1, 2, 4, and 6 days after injection of radiolabeled antibody, five mice at each time point were euthanized and blood was obtained from the heart. Tumors and major organs were removed and weighed, and radioactivity counts were measured using a gamma counter (PerkinElmer). The data were expressed as the percentage of injected dose per gram of tissue (% ID/g). Data are expressed as mean ± SD. Tumor uptake data were analyzed by ANOVA with the Student–Newman–Keuls method multiple comparison test.

### PET/computed tomography (CT) imaging

We conducted imaging in two mice bearing subcutaneous tumors (MIA Paca-2 and A4) with approximately 10 mm in diameter and one mouse bearing an orthotopic tumor (MIA Paca-2 alone) 3 weeks after implantation. Mice were injected with approximately 3.7 MBq of ^89^Zr-labeled antibody into the tail vein. The injected protein dose was adjusted to 100 µg per mouse by the addition of unlabeled antibody. PET data acquisition was conducted at 30 min, and 1, 2, 4 and 6 days after administration for 10 to 20 min using a small-animal PET system (Inveon, Siemens Medical Solutions, Malvern, PA, USA) under isoflurane anesthesia. Body temperature was maintained at 36°C to 37°C by a lamp and a heating pad during the scan. Images were reconstructed using a 3D maximum *a posteriori* (18 iterations with 16 subsets, β = 0.2) without attenuation correction. Tracer uptake was expressed as % ID/g. The region of interest was manually drawn over tumors and tracer uptake was quantified using ASI Pro software (Siemens Medical Solutions). After PET scanning of an orthotopic tumor mouse model, CT images were acquired with an X-ray source set at 90 kVp and 200 µA using a small-animal CT system (R_mCT2, Rigaku, Tokyo, Japan). After imaging, we conducted biodistribution experiments to confirm PET results.

## Results

### CD147 protein expression in pancreatic cancer cells and xenografts

**Figure 1:**
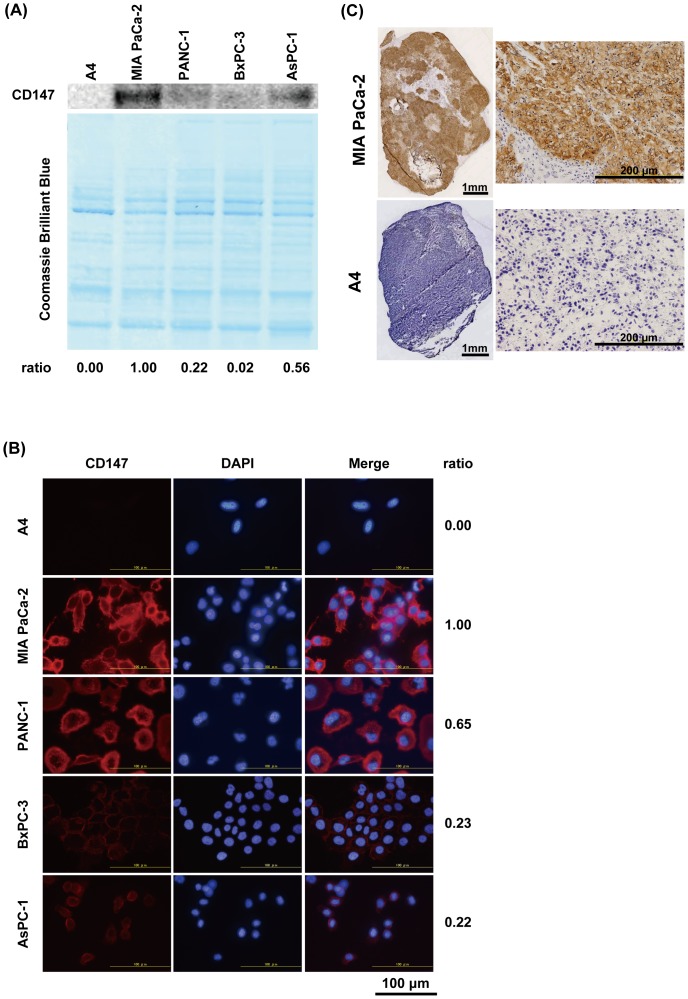
CD147 protein expression analysis of pancreatic cancer cell lines (MIA Paca-2, PANC-1, BxPC-3, and AsPC-1) and A4 as a negative control. (A) Western blotting analysis of total cell lysate using anti-CD147 antibody (upper panel) and Coomassie Brilliant Blue staining of the same PVDF membrane as a loading control (lower panel). The ratio of band intensity is shown under the panels. (B) Subcellular localization of CD147 protein determined by immunofluorescence staining with anti-CD147 antibody (red) and DAPI nucleic acid staining (blue). The ratio of CD147 intensity is shown on the right of the panels. (C) CD147 expression in MIA Paca-2 (upper) and A4 (lower) xenografted tumors determined by immunohistochemical staining of frozen sections (10 µm thick).

We determined CD147 protein expression in four human pancreatic cancer cell lines (MIA Paca-2, PANC-1, BxPC-3, and AsPC-1) and the mouse A4 cell line as a negative control, using western blotting and immunofluorescence staining. CD147 expressed in the four human pancreatic cancer cell lines, but not in A4 (Figure 1A and B). MIA Paca-2 showed the highest expression, followed by AsPC-1, PANC-1, and BxPC-3 as determined by western blotting analysis (Figure 1A). MIA Paca-2 also showed the highest expression as determined by immunofluorescence staining, but expression in AsPC-1, which was second highest in western blotting, was lower than that of PANC-1 (Figure 1B). CD147 protein was mainly localized on the plasma membrane of CD147-expressing cells (Figure 1B). CD147 expression was not detected in A4 cells by western blotting or immunofluorescence analyses. Next, we conducted CD147 immunohistochemical staining of MIA Paca-2 and A4 subcutaneous tumors. In MIA Paca-2 tumors, viable cancer cells were intensely stained, but not necrotic cells or stroma (Figure 1C upper panel). No protein expression was detected in A4 tumors (Figure 1C lower panel).

### *In vitro* characterization of radiolabeled 059-053

**Figure 2:**
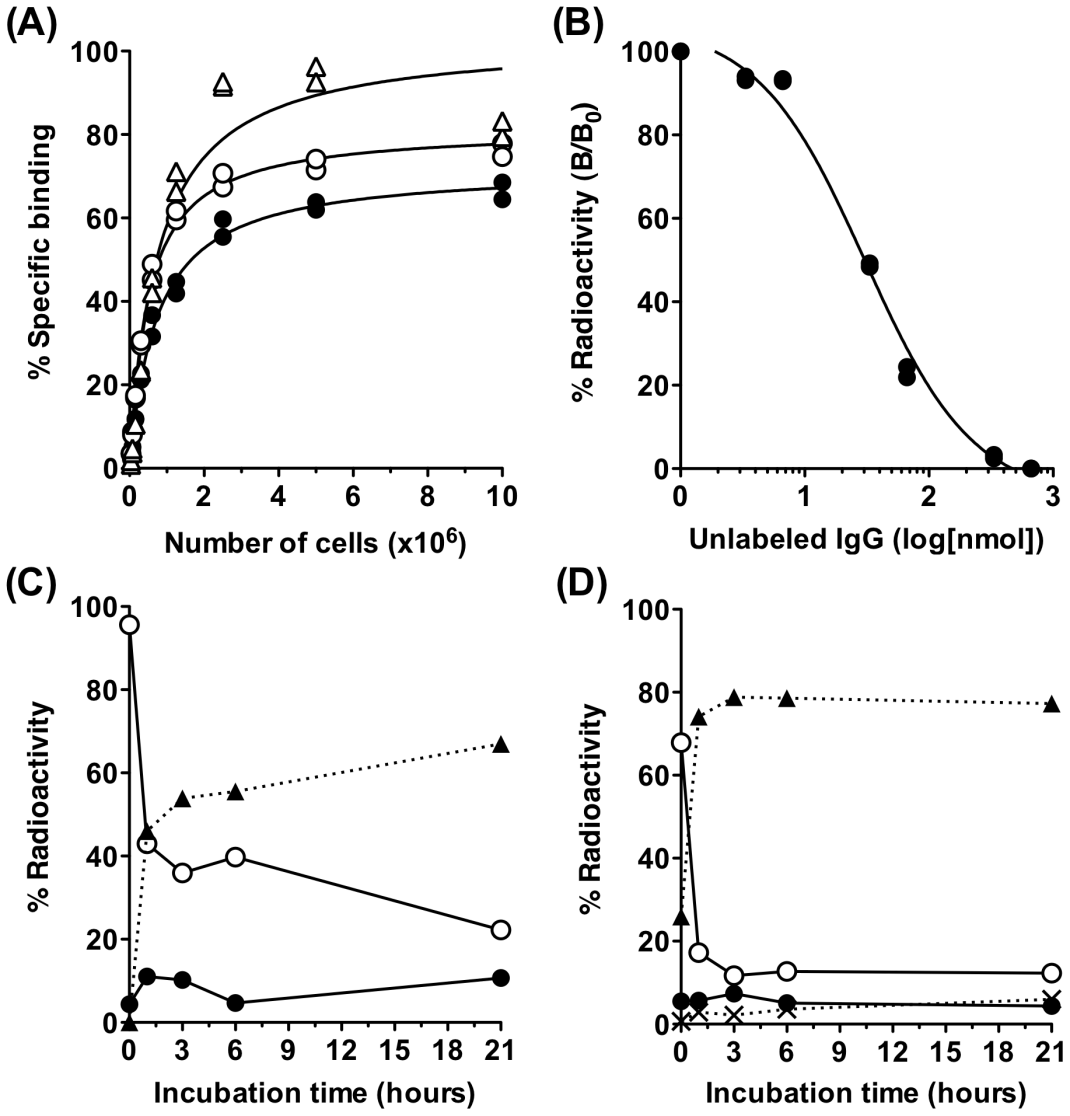
*In vitro* assay of radiolabeled anti-CD147 antibody 059-053 using MIA PaCa-2 cells. (**A**) Cell binding assay for [^89^Zr]059-053 (white triangles), [^67^Ga]059-053 (white circles) and [^125^I]059-053 (black circles). (**B**) Competitive inhibition assay for [^125^I]059-053 (black circles). Internalization assay for [^67^Ga]059-053 (**C**) and [^125^I]059-053 (**D**). Changes in % of total radioactivity for each fraction are plotted against incubation time at 37°C (black circles, internalized fraction; white circles, membrane-bound fraction; black triangles, protein-bound fraction in the culture medium; cross marks, non-protein-bound fraction in the culture medium). These assays were conducted in duplicate. Data represent each replicate in A and B and mean in C.

We labeled human anti-CD147 monoclonal antibody 059-053 with ^125^I, ^67^Ga, or ^89^Zr and conducted a cell binding assay. Binding of [^125^I]059-053, [^67^Ga]059-053, and [^89^Zr]059-053 at 5×10^6^ MIA Paca-2 cells was 63%, 73%, and 94%, respectively (Figure 2A). However, binding of [^125^I]059-053 to 5×10^6^ A4 cells was only 1.3% (data not shown). The immunoreactive fraction of [^125^I]059-053, [^67^Ga]059-053, and [^89^Zr]059-053 in MIA Paca-2 cells was estimated to be 0.80, 0.96, and 1.00, respectively. The equilibrium dissociation constant and binding site of 059-053 was estimated to be 15.3 nM and 5.4×10^4^ sites per MIA Paca-2 cell, respectively, by competitive inhibition assay (Figure 2B). We examined the temporal change in radioactivity localization of [^67^Ga]059-053 and [^125^I]059-053 in MIA Paca-2 cells. The cell membrane-bound fraction rapidly decreased, whereas the protein-bound fraction in the culture medium rapidly increased for both radiolabeled antibodies (Figure 2C and D). Approximately 10% of [^67^Ga]059-053 at maximum was internalized after incubation (Figure 2C). When cells were incubated on ice, the membrane-bound fraction did not change for at least 3 h (data not shown).

### *In vivo* biodistribution of radiolabeled 059-053

**Figure 3:**
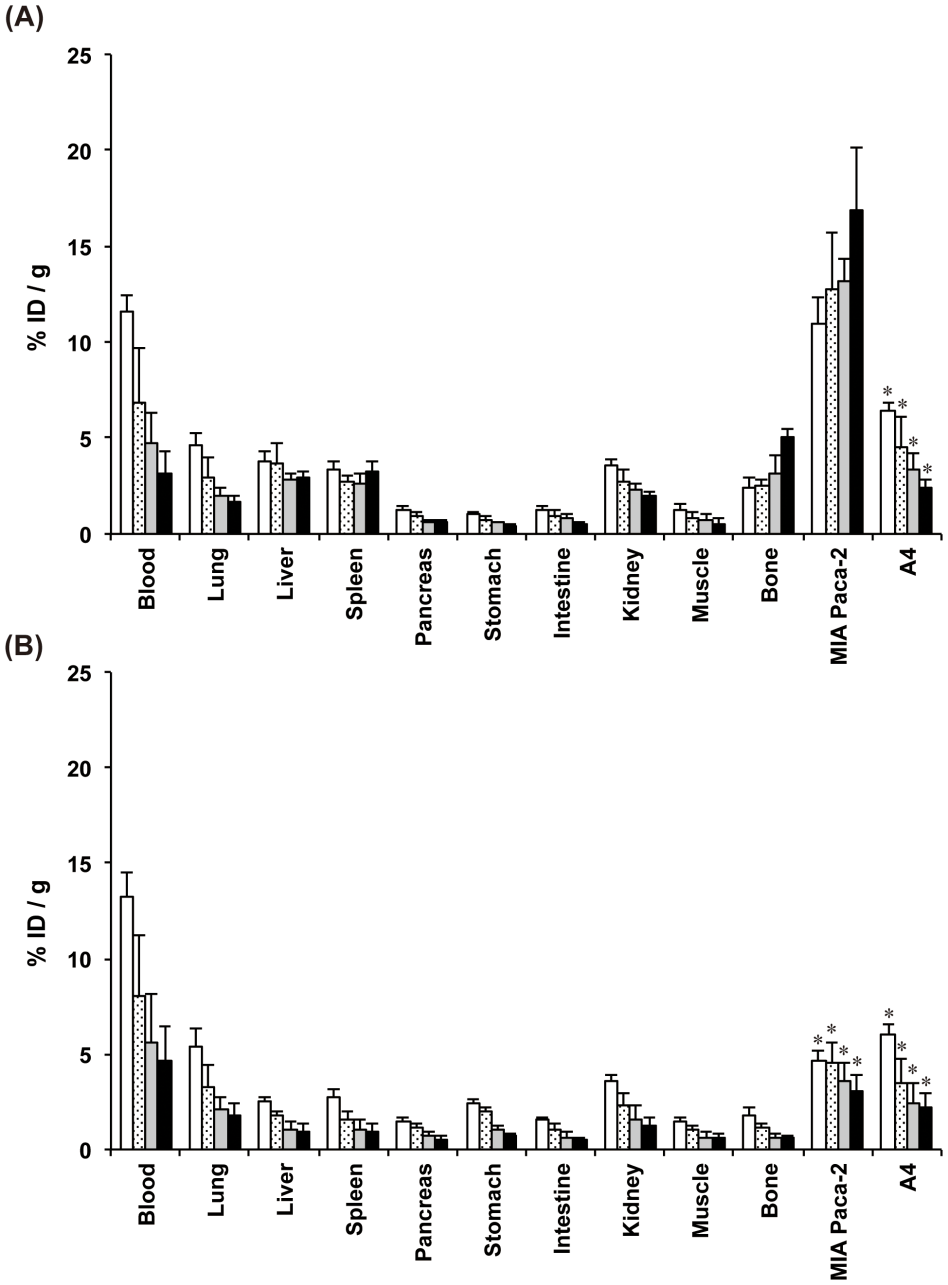
*In vivo* biodistribution experiments in nude mice bearing MIA PaCa-2 and A4 xenografts of radiolabeled anti-CD147 antibody 059-053. Samples were collected and weighted, and radioactivity was measured at day 1 (white bars), 2 (dot bars), 4 (gray bars) and 6 (black bars) after intravenous injection of 37 kBq each of [^89^Zr]059-053 (A) and [^125^I]059-053 (B). Data are expressed as mean ± SD (n = 5). *P<0.01 vs. [^89^Zr]059-053 tumor uptake at each time point analyzed by ANOVA with the Student–Newman–Keuls method multiple comparison test.

Biodistribution experiments using ^89^Zr- and ^125^I-labeled 059-053 were conducted in nude mice bearing both MIA Paca-2 and A4 xenograft tumors (n = 5 at each time point). [^89^Zr]059-053 uptake in MIA Paca-2 tumors was 11.0±1.3% ID/g at day 1, which increased with time reaching 16.9±3.2% ID/g at day 6 (Figure 3A). In contrast, uptake in A4 tumors (non-CD147-expressing) was low and decreased with time (Figure 3A). MIA Paca-2-to-A4 uptake ratio of [^89^Zr]059-053 was 1.7±0.2 at day 1 and increased with time (7.1±0.5 at day 6). The uptake of [^89^Zr]059-053 in normal major organs including the pancreas was low and decreased gradually with time except for bone (Figure 3A). The tumor-to-pancreas uptake ratio of [^89^Zr]059-053 was 9.1±1.5 at day 1 in MIA Paca-2 tumors, and it increased to 30.0±6.7 at day 6. MIA Paca-2 tumor uptake of [^125^I]059-053 was 4.6±0.5% ID/g at day 1 and persisted until day 2, (4.6±1.6% ID/g), but decreased thereafter (Figure 3B). The uptake in normal major organs of [^125^I]059-053 decreased gradually with time including bone (Figure 3B). [^89^Zr]059-053 uptake in MIA Paca-2 tumors at all time points was significantly higher compared with that in A4 tumors and [^125^I]059-053 uptake in MIA Paca-2 and A4 tumors (*P*<0.01).

### PET imaging of tumor-bearing mice with [^89^Zr]059-053

**Figure 4:**
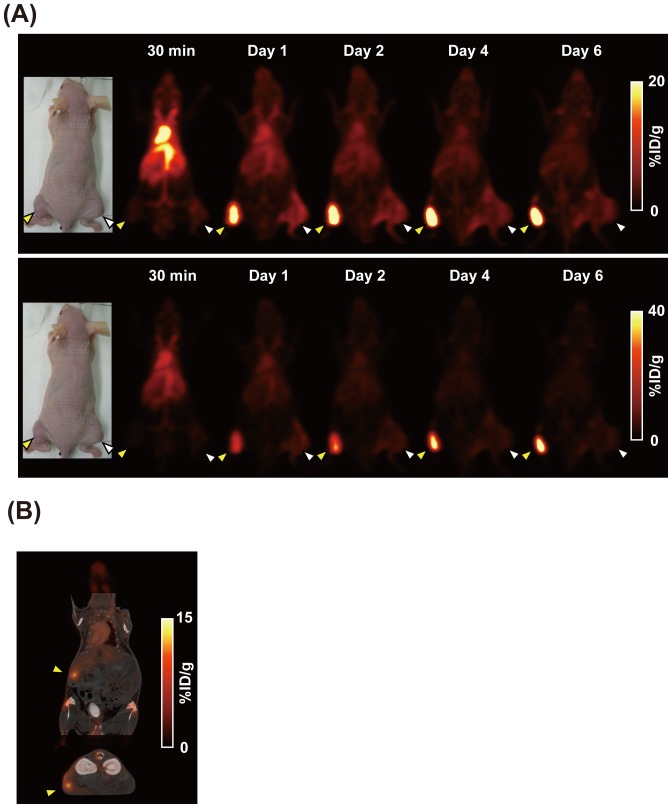
PET imaging with ^89^Zr-lableled anti-CD147 antibody 059-053. (**A**) Serial PET images (maximum-intensity-projection) of a nude mouse bearing MIA PaCa-2 (yellow arrowhead) and A4 (white arrowhead) xenografted tumors at 30 min, and days 1, 2, 4, and 6 after intravenous injection of 3.7 MBq [^89^Zr]059-053. PET images of the same mouse are shown at different scale settings. (**B**) Coronal (upper) and transaxial (lower) images of PET/CT in the mouse orthotopic pancreatic cancer model (yellow arrowhead, MIA Paca-2) at day 6 after injection.

To confirm the result of the biodistribution study, we conducted PET imaging with [^89^Zr]059-053. Serial PET images in two subcutaneous tumor models bearing MIA Paca-2 and A4 tumors were obtained at 30 min, and days 1, 2, 4, and 6 after injection. At 30 min, radioactivity in the blood pool was very high, whereas that in MIA Paca-2 and A4 tumors was low with no difference between the two (Figure 4A). At day 1, uptake in MIA Paca-2 tumors (10.8% ID/g) was markedly increased and higher than in the A4 tumors (6.6% ID/g), and the background activity was decreased. The MIA Paca-2 tumor uptake further increased from 12.2% ID/g at day 2 to 17.5% ID/g at day 6, while the background activity continued to decrease, resulting in an increase in the contrast of MIA Paca-2 tumors over time (Figure 4A). A4 tumor uptake gradually decreased from 6.6% ID/g at day 1 to 4.1% ID/g at day 6. These data were basically consistent with results in the biodistribution study. PET/CT images in the orthotopic tumor model obtained at day 6 showed that PET with [^89^Zr]059-053 could visualize the orthotopic implanted tumor located in the pancreatic tail (Figure 4B). From PET data in the orthotopic model, tumor uptake of [^89^Zr]059-053 was 8.6% ID/g at day 6. From biodistribution data after PET, [^89^Zr]059-053 uptake in the same orthotopic tumor was 15.5% ID/g. Since the orthotopic tumor size was approximately 5 mm in diameter, the partial volume effect could affect quantitative data from PET.

## Discussion

Pancreatic cancer is one of the most lethal human malignancies, ranking fifth and fourth among cancer-related death in men and women, respectively, in economically developed countries [1]. Its prognosis is very poor and the 5-year survival rate for patients in the United States with localized and distant metastatic cancer is 22% and 2%, respectively [2]. Therefore, additional effective anticancer therapy is required, especially for patients with metastatic cancer. CD147 highly expresses in pancreatic cancer [4] and is involved in the metastatic process [3], and therefore is considered a good candidate for targeted cancer therapy [3]. CD147-specific noninvasive imaging could be useful to select appropriate patients most likely to benefit from anti-CD147 therapy. In the present study, we evaluated the *in vitro* and *in vivo* properties of a novel fully human monoclonal antibody 059-053 that recognizes CD147, and labeled it with a positron emitter ^89^Zr, to develop a new PET probe to detect CD147 expression in pancreatic cancer.

First, we evaluated CD147 protein expression of four pancreatic cancer cell lines (MIA Paca-2, PANC-1, BxPC-3, and AsPC-1) by western blotting and immunofluorescence staining to select a suitable pancreatic cancer cell line to assess radiolabeled 059-053. The MIA Paca-2 cell line showed the highest expression as determined by western blotting and immunofluorescence staining analysis. In addition, we confirmed that MIA Paca-2 cells formed subcutaneous and orthotopic tumors in nude mice and high CD147 expression in these tumors was determined by immunohistochemical staining. A4 cells showed no CD147 protein expression either *in vitro* or *in vivo*. Therefore, we chose MIA Paca-2 cells as a positive control and A4 cells as a negative control for the following evaluation.

Next, we radiolabeled 059-053 with ^125^I, ^67^Ga, or ^89^Zr and evaluated its *in vitro* properties. Cell binding and competitive inhibition assays revealed that 059-053 specifically bound to MIA Paca-2 cells with high affinity, but not to A4 cells. The immunoreactive fraction of radiolabeled antibodies was more than 0.8, indicating that the loss of immunoreactivity by radiolabeling procedures was minimal. The internalization assay showed that protein-bound fractions in the culture medium rapidly increased after incubation at 37°C, and the internalized fraction was low. This suggests that CD147–antibody complex is easily detached from the plasma membrane under the conditions/procedures of this internalization experiment. In the present study, although we employed ^67^Ga on behalf of ^89^Zr for the internalization assay, this result is considered to be consistent with that using ^89^Zr-labeled antibody because the chelate (DF) is common between two radiometals, these radiometals with DF complex is known to be stable *in vitro* and *in vivo* [18], [19], [20], [21], [22], and biodistribution was reported to be similar between ^68^Ga- and ^89^Zr-labeled antibody [20]. In contrast to the result of internalization assay, the *in vivo* distribution study demonstrated that uptake of [^89^Zr]059-053 in MIA Paca-2 tumors was very high and increased with time, suggesting that the CD147–antibody complex did not detach from the membrane *in vivo*. Furthermore, a difference in tumor uptake patterns between [^89^Zr]059-053 and [^125^I]059-053 strongly suggests that radiolabeled 059-053 was internalized in cells after binding to CD147 on the cell surface *in vivo*. After metabolism inside the cells, ^125^I activity would be cleared from cells, while ^89^Zr activity would be retained in cells. Taken together, trypsinization of cells probably caused the results we observed in the internalization assay, although the duration of trypsinization was made as short as possible to minimize damage to CD147 on the cell surface by trypsin. In the present case, the *in vivo* distribution was not fully recapitulated by the *in vitro* experiments. It is noteworthy that the *in vivo* study with subcutaneous xenografts demonstrated that [^89^Zr]059-053 highly accumulated in MIA Paca-2 tumors, but not in A4 tumors. This was confirmed by serial PET imaging, indicating that [^89^Zr]059-053 is a promising PET probe for the detection of CD147-expressing tumors and in the selection of appropriate patients for anti-CD147 therapy. Biodistribution studies have shown that the uptake of [^89^Zr]059-053 in normal major organs decreased with time except for bone, which is not likely to be actual antibody uptake, but bone-seeking ^89^Zr catabolites just the same as for other antigens [21], [22], [23], [24]. Since our anti-CD147 antibody 059-053 does not bind to mouse CD147, our results in the murine model may not fully predict the distribution in human patients. CD147 expression is reported to be high in most cancer tissues, but it is limited in normal tissues [4]. Scintigraphy with ^131^I-labeled anti-CD147 F(ab')_2_ in patients with hepatocellular carcinoma (HCC) has shown lower uptake in normal organs than in HCC tissues [25]. Therefore, our anti-CD147 antibody 059-053 is expected to show low accumulation in normal organs of patients, although further clinical study will be necessary to precisely evaluate normal organ uptake.

Subcutaneous tumor models are powerful tools for oncological investigations, but these models cannot always mimic clinical findings. For example, the tumor microenvironment influences cell migration such that orthotopic xenografts give rise to a higher incidence of metastatic lesions than do subcutaneous xenografts [11], [26]. Next, we conducted PET/CT imaging in the orthotopic pancreatic tumor mouse model and demonstrated that [^89^Zr]059-053 visualized orthotopically implanted tumors. We evaluated tumor uptake using only the CD147-positive tumor and did not conduct blocking experiments or imaging using control antibodies in the orthotopic tumor model. Therefore, there may be a risk, although low, it is worth considering, that the uptake was not antigen specific. However, considering the results of biodistribution and PET studies using both CD147-positive and negative tumors in the subcutaneous tumor models, high uptake in the orthotopic tumor is likely to be antigen specific rather than non-specific. Thus, [^89^Zr]059-053 could be useful for studies investigating CD147-targeted therapy efficacy or metastatic process in orthotopic pancreatic cancer models.

With regard to the imaging of CD147 expression by means of radiolabeled antibody, two CD147-targeted single-photon emission computed tomography (SPECT) imaging probes, ^131^I-labeled anti-CD147 F(ab')_2_ (Licartin) and ^99m^Tc-labeled anti-CD147 IgG ([^99m^Tc]IgG), have been reported to date [6], [27]. In the former, Licartin visualized HCC in patients at day 7 after injection. ^131^I emits both γ-ray and β-particles, and can be used for scintigraphic imaging and radioimmunotherapy. For imaging purposes, γ-rays emitted from ^131^I are too high and not optimal. Preclinical and clinical studies of Licartin have been conducted with the major purpose of developing an anticancer therapy [6], [7], [28]. In the latter study, the [^99m^Tc]IgG visualized an orthotopic MIA Paca-2 tumor 4 h after injection [27]. A long half-life of IgG in blood (110 h) results in an optimal imaging time of 2 to 4 days after injection [29]. Therefore, gamma- and positron-emitters that have a longer physical half-life (T_1/2_), such as ^111^In (T_1/2_ = 67.3 h), ^67^Ga (T_1/2_ = 78.2 h), ^89^Zr (T_1/2_ = 78.4 h), and ^124^I (T_1/2_ = 100.2 h), are desirable for the application of IgG to nuclear medicine imaging. This indicates that T_1/2_ of ^99m^Tc (6.0 h) is not suitable for IgG-based imaging. Compared with γ-ray imaging, PET has higher sensitivity and quantification ability than SPECT and could be suitable for detecting small-sized metastatic tumors. Since Licartin is a human–mouse chimeric antibody [6] and the [^99m^Tc]IgG is a mouse antibody [27], both antibodies have mouse-derived portions. In contrast, 059-053 is a fully human antibody and has no non-human-derived proteins, and is therefore expected to be less immunogenic. Taken together, PET with [^89^Zr]059-053 can potentially detect CD147-expressing tumors with high sensitivity and reduced toxicity in cancer patients. Furthermore, 059-053 has ADCC activity and the potential to be used for CD147-targeted therapy, including radioimmunotherapy.

CD147 highly expresses not only in pancreatic cancer but also in many other types of cancers, such as glioma, ovarian cancer, renal cell carcinomas, bladder carcinoma, and hepatocellular carcinoma [3], [4]. Moreover, its increased expression is associated with poor prognosis in several cancers, such as breast, cervical, liver, colorectal, and bladder cancers, and it is considered to be caused by increased metastatic rates and treatment resistance [3], [30], [31], [32]. In the present study, we showed that [^89^Zr]059-053 uptake in major organs was low. Taken together, [^89^Zr]059-053 could detect CD147-expressing tumors in the pancreas but also in other organs, and it could be used to assess the metastatic potential and treatment resistance of these tumors, although further studies will be necessary to prove these potential uses.

## Conclusion

We radiolabeled a fully human anti-CD147 monoclonal antibody 059-053 and evaluated its *in vitro* and *in vivo* properties for use as a new CD147-targeted PET imaging probe in a model of pancreatic cancer. [^89^Zr]059-053 highly accumulated in CD147-expressing tumors and clearly visualized subcutaneously and orthotopically implanted xenografts. PET with [^89^Zr]059-053 is a promising noninvasive imaging method to provide useful information for the selection of appropriate cancer patients who could gain benefit from CD147-targeted therapy, although further clinical studies are necessary.
